# Supplementary material for: Responses of Humoral and Cellular Immune Mediators in BALB/c Mice to LipX (PE11) as Seed Tuberculosis Vaccine Candidates
Source: Genes (Basel). 2022 Oct 26;13(11):1954. doi: 10.3390/genes13111954 (PMC9690253; doi:10.3390/genes13111954)
Supplement: Supplementary file 1 [file genes-13-01954-s001.zip › genes-1934357-supplementary.pdf]

Supplementary material

Table S1. Comparison of cytokine production induced by LipX-His or PHA or without induction in the pcDNA3.1-*lipX*-immunized group.

Cytokines	Induction			*p*-value
	LipX-His	PHA	No induction	
IL-4	59.88 + 8.32	39.44 + 7.89	32.02 + 2.50	0.000
IL-10	23.86 + 6.77	43.57 + 6.49	18.71 + 11.09	0.232
IL-12	221.14 ± 46.23	54.86 ± 13.98	52.29 ± 11.93	0.000
IFN-γ	441.22 ± 54.55	307.22 ± 51.77	258.56 ± 16.43	0.000
